# Internet-based cognitive therapy for women with antenatal depressive symptoms during the COVID-19 pandemic: protocol for a multi-center randomized controlled trial across China

**DOI:** 10.1186/s13063-022-06728-5

**Published:** 2022-09-21

**Authors:** Chen-Chi Duan, Jia-Le Yu, Jing Tao, Chen Zhang, Dan Zhang, Xiu Zeng, Wan-Ting Zeng, Hua-Lin Xu, Jian-Yin Qiu, Cindy-Lee Dennis, Li Jin, He-Feng Huang, Yan-Ting Wu

**Affiliations:** 1 Obstetrics and Gynecology Hospital, Institute of Reproduction and Development, Fudan University, 128 Shenyang Road, Yangpu Districtz, Shanghai, 200011 China; 2 Research Units of Embryo Original Diseases, Chinese Academy of Medical Sciences (No. 2019RU056), Shanghai, China; 3 International Peace Maternity and Child Health Hospital, Shanghai Jiao Tong University School of Medicine, Shanghai, China; 4 Shanghai Mental Health Center, School of Medicine, Shanghai Jiao Tong University School of Medicine, Shanghai, China; 5 Key Laboratory of Reproductive Genetics and Department of Reproductive Endocrinology, Women’s Hospital, Zhejiang University School of Medicine, Hangzhou, Zhejiang China; 6 Hunan Maternal and Child Health Care Hospital, Changsha, Hunan China; 7 Hangzhou Women’s Hospital, Hangzhou, Zhejiang China; 8 Shaoxing Maternity and Child Health Care Hospital, Shaoxing, Zhejiang China; 9 Lawrence S. Bloomberg Faculty of Nursing, University of Toronto, Toronto, Ontario Canada

**Keywords:** Antenatal depression, Cognitive behavior therapy, Internet, Psychological distress, Anxiety, COVID-19

## Abstract

**Background:**

Depression and anxiety are common among pregnant women. Internet-delivered psychological therapies such as cognitive behavioral therapy (iCBT) have been developed to increase accessibility and address common help-seeking barriers, especially during pandemic period. The objective of this trial is to evaluate the short-term and long-term effects of iCBT on reducing depressive symptoms among pregnant women during the COVID-19 pandemic with the overall goal of preventing depression recurrence in the first 12 months postpartum.

**Methods:**

A multi-site randomized controlled trial will be conducted where 300 pregnant women early in their third trimester will be screened for depression symptoms using the Edinburgh Postnatal Depression Scale (EPDS) during a routine obstetrical visit. Eligible and consenting women with a score greater than 9 will be randomly allocated (1:1) to either intervention group or control group. ICBT involving the completion of 7 weekly online modules will be delivered via a well-designed perinatal mental healthcare app. The primary objective is to evaluate the effect of iCBT on reducing depression symptoms among pregnant Chinese women starting from their third trimester. The secondary objectives are to examine the effect of iCBT on anxiety, sleep quality, social support, parenting stress, co-parenting relationship, and infant development.

**Discussion:**

This multi-center randomized controlled trial has been planned in accordance with best practices in behavioral trial design. The internet-based intervention addressed the needs of pregnant women during a major pandemic where face-to-face therapy is not preferable. The trial has a relatively large sample size with sufficient power to evaluate the efficacy of iCBT intervention for the primary and secondary outcomes. One year follow-up evaluation in the study is designed to determine the longer-term effect of the intervention on both maternal and infant outcomes. Although a limitation is the assessment of depression and anxiety using self-report measures, these easily incorporated and maternal-preferred assessments allow for real-life scalability if the intervention is proven to be effective.

**Ethics and dissemination:**

Ethics was approved by the institutional review board of International Peace Maternity and Child Health Hospital (GKLW2020-25). Dissemination of results will be published in peer-reviewed academic journals and presented at scientific conferences.

**Trial status:**

The first patient was enrolled on 19 August 2020. To date, 203 participants have met eligibility requirements and been randomized to either the intervention group or control group. Data collection aims to be complete in September 2022. Date and version identifier: 2020715-version1.0.

**Trial registration:**

ChiCTR2000033433. Registered 31 May 2020, http://www.chictr.org.cn/showproj.aspx?proj=54482.

**Supplementary Information:**

The online version contains supplementary material available at 10.1186/s13063-022-06728-5.

## Introduction

Large-scale infectious diseases are known to have adverse psychological effects on both the general population and specific vulnerable subgroups [1–4]. During the coronavirus disease 2019 (COVID-19) pandemic, frequently reported concerns such as fear of infection, social isolation, stigmatization, discrimination, unemployment, and financial loss have resulted in a significant increase in mental illness rates among women and younger populations [5]. In a cohort study spanning China, pregnant women after the declaration of COVID-19 human-to-human transmission in China were significantly more likely to have depression and thoughts of self-harm than pregnant women before the pandemic [6].

Maternal depression and anxiety are the most common psychiatric disorders that develop during pregnancy and postpartum [7]. Although reports of mental illness related to the perinatal period date back to the middle ages, the management of this global public health issue remains suboptimal. Postpartum depression prevalence rates vary among countries, ranging from 6.9 to 12.9% in high-income countries to more than 20% in some low- or middle-income countries [8]. Whether the incidence of depression peaks postnatally is questionable given research showing that 33% of women with depression actually developed symptoms during pregnancy [9]. Anxiety is equally common as depression postnatally with meta-analytic data suggesting 15% of women have high levels of anxiety across the first year postpartum and that 9% of women develop comorbid depression and anxiety. The strongest risk factor for postpartum depression or anxiety is a history of a mental illness, especially during pregnancy. Most depression or anxiety episodes resolve within a few months of treatment, but one in four women diagnosed with depression are still symptomatic at one year postpartum and about 40% will relapse [10]. Importantly, antenatal depression is associated with adverse pregnancy outcomes including elective or emergency cesarean section, preterm birth, and small for gestational age [11–14]. It is also well documented that postpartum depression has consequences for the mother and her family with broader negative effects related to work, caregiving, and society as well. Moreover, one particular concern is the influence of maternal depression on parenting behaviors, which results in increased risk of poor maternal-child attachment and, in the longer term, impaired emotional, social, and cognitive development [15] including internalizing and externalizing psychopathology [16].

Although the far reaching adverse effects of perinatal mental illness is internationally recognized, it surprisingly continues to be under-treated. In many countries, perinatal mental health services in antenatal care remain limited due to women’s reluctance to report changes in their mood [17] and provider barriers such as lack of time, limited knowledge, and cost/insurance mismatch [18]. During the COVID-19 epidemic, there is an even greater need for perinatal mental health services including the provision of easily accessible preventive and treatment interventions [19, 20].

Psychological therapies are the first-line treatment recommended for adults with mild to moderate depression [21]. Cognitive behavioral therapy (CBT) is a practical, short-term form of psychotherapy where individuals learn to identify, question, and change the thoughts, attitudes, and beliefs related to the negative emotional and behavioral reactions. It is a highly effective depression treatment among the general population and perinatal women [22]. Moreover, internet-based programs have been developed to increase accessibility and address common help-seeking barriers [23]. While evidence for the effectiveness of iCBT on the treatment of depression is encouraging [24], not all studies have demonstrated an improvement in symptomatology compared to inactive controls [25]. There is also evidence showing that CBT can be initiated in pregnancy for the prevention of postpartum depression [26]; however, no studies have examined the effect of iCBT on women at high risks of antenatal depression during a large-scale infectious diseases outbreak where limited medical resources are provided. To meet the urgent need for reliable access to effective depression interventions during the COVID-19 outbreak and to develop a new collaborative initiative between the departments of obstetrics and psychiatry in China, the purpose of the proposed trial is to evaluate the effect of iCBT on treating depression symptoms among pregnant women with the aim of preventing postpartum depression.

### Study objectives

The primary objective of this study is to evaluate the effect of iCBT on reducing depression symptoms among pregnant women in their third trimester with the aim of preventing depression across the postpartum period assessed using the Patient Health Questionnaire 9-item scale (PHQ-9) [27]. The secondary objectives are to evaluate the effect of iCBT on (1) anxiety using the Generalized Anxiety Disorder (GAD-7) [28], (2) sleep quality using the Pittsburgh Sleep Quality Index (PSQI) [29], (3) social support using the Multidimensional Scale of Perceived Social Support (MSPSS) [30], (4) parenting stress using Parenting Stress Index-Short Form (PSI-SF) [31], (5) co-parenting relationship using the Brief Co-parenting Relationship Scale (BCRS) [32], and (6) infant development using the Denver Development Screen Test (DDST) [33].

## Methods and analysis

### Study design

**Fig. 1:**
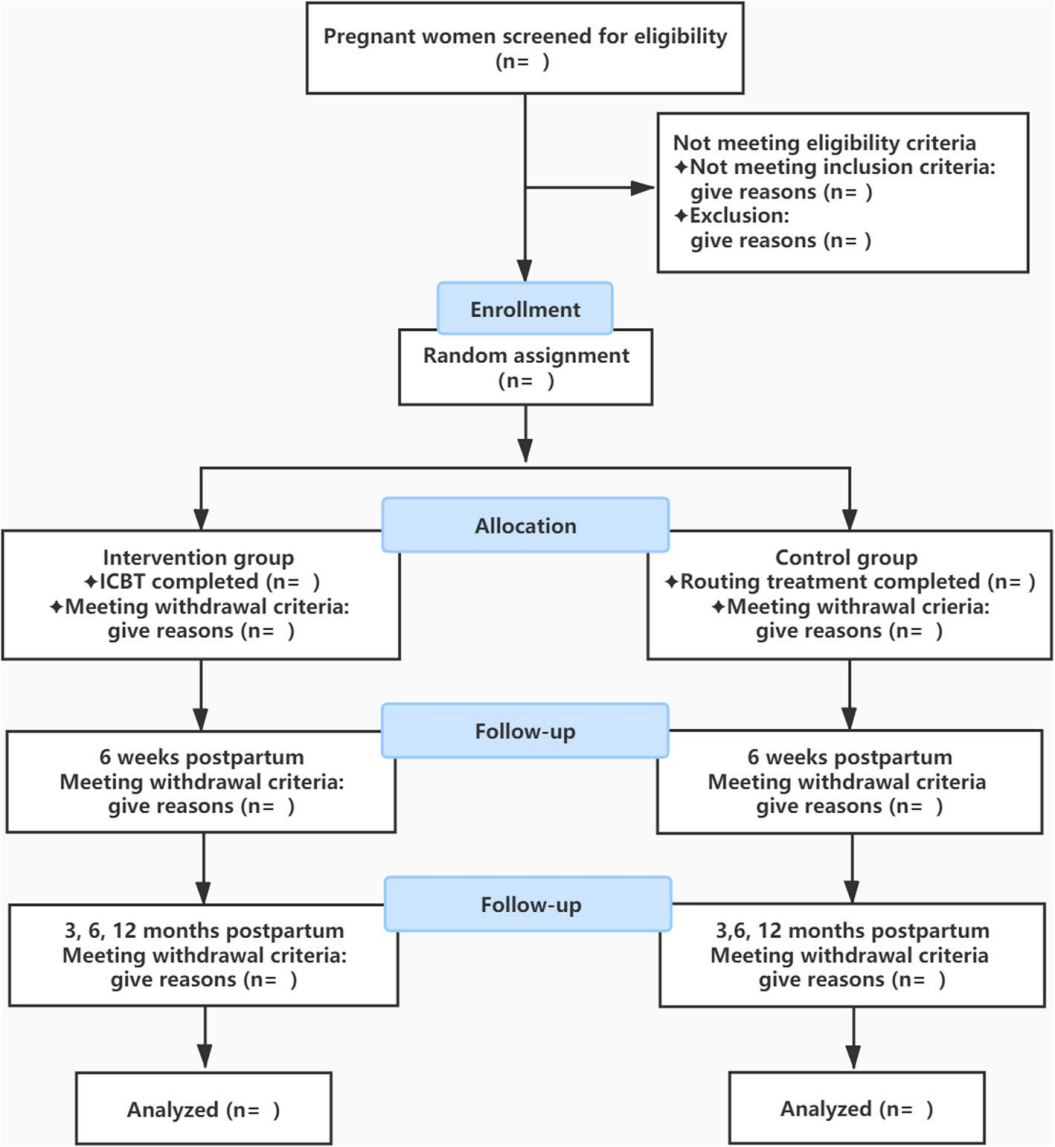
Flowchart of the study design

The study is a multi-center, superiority, randomized controlled trial of two parallel groups that adheres to CONSORT guidelines [34]. Figure 1 depicts a flow diagram of the study design.

### Study setting

Pregnant women will be recruited through obstetric clinics at the International Peace Maternity and Child Health Hospital in Shanghai China and other four cooperative medical centers from different regions in China including (1) Hunan Maternal and Child Health Care Hospital in Changsha, Hunan; (2) Women’s Hospital affiliated with Zhejiang University School of Medicine in Hangzhou, Zhejiang Province; (3) Hanghzhou Women’s Hospital in Hangzhou, Zhejiang Province; and (4) Shaoxing Maternity and Child Health Care Hospital in Shaoxing, Zhejiang Province.

### Participant eligibility

#### Inclusion criteria

All pregnant women are eligible to participate if they meet the following inclusion criteria: (1) singleton pregnancy; (2) ≥ 28 weeks gestation; (3) 18 years of age or older; (4) able to read and understand Chinese; (5) has an Edinburgh Postnatal Depression (EPDS) [35] score > 9; and (6) has reliable internet access via a smartphone or computer.

#### Exclusion criteria

Exclusion criteria include (1) active suicidal ideation, (2) severe psychopathology (e.g., schizophrenia), and (3) currently receiving treatment for depression and anxiety including other psychotherapy or medication.

### Procedure

The study procedure is described by following the Standard Protocol Items: Recommendations for Interventional Trials (SPIRIT) checklist (Supplementary Material).

#### Participant screening, recruitment, and enrolment

All pregnant women will be assessed with EPDS by a trained research assistant during their standard antenatal visit between 28 and 34 weeks gestation. Those with an EPDS > 9 will be introduced to the study and provided with a detailed study explanation. Eligible women agreeing to participate will complete informed consent procedures and will be financially compensated when they finished the trial. Following the completion of a baseline questionnaire, women will be randomized to either the control group (standard care for women with antenatal depressive symptoms) or the intervention group (standard care for women with antenatal depressive symptoms plus access to iCBT).

#### Randomization and blinding

Following the collection of baseline data, eligible women will be allocated in 1:1 ratio to either the intervention group or control group by the research assistant using a computer-generated block randomization procedure provided by the Clinical Research Public Technical Service Platform (http://www.scrcnet.org/CRIS_en.asp#top). The allocation sequence will be implemented by using a web-based central randomization system. Participants will then be assigned to either the intervention group or the control group according to the results of the computer-generated randomization system. Due to the nature of the intervention, health care professionals providing iCBT intervention will not be blinded to group allocation but participants will be. Follow-up questionnaires will be completed confidentially online by participants and depression scores will be reviewed by a trained research assistant not involved in other trial activities. Safety protocols used in previous trials completed by team members will be followed for participants who score positively to self-harm ideation.

#### Intervention

**Fig. 2:**
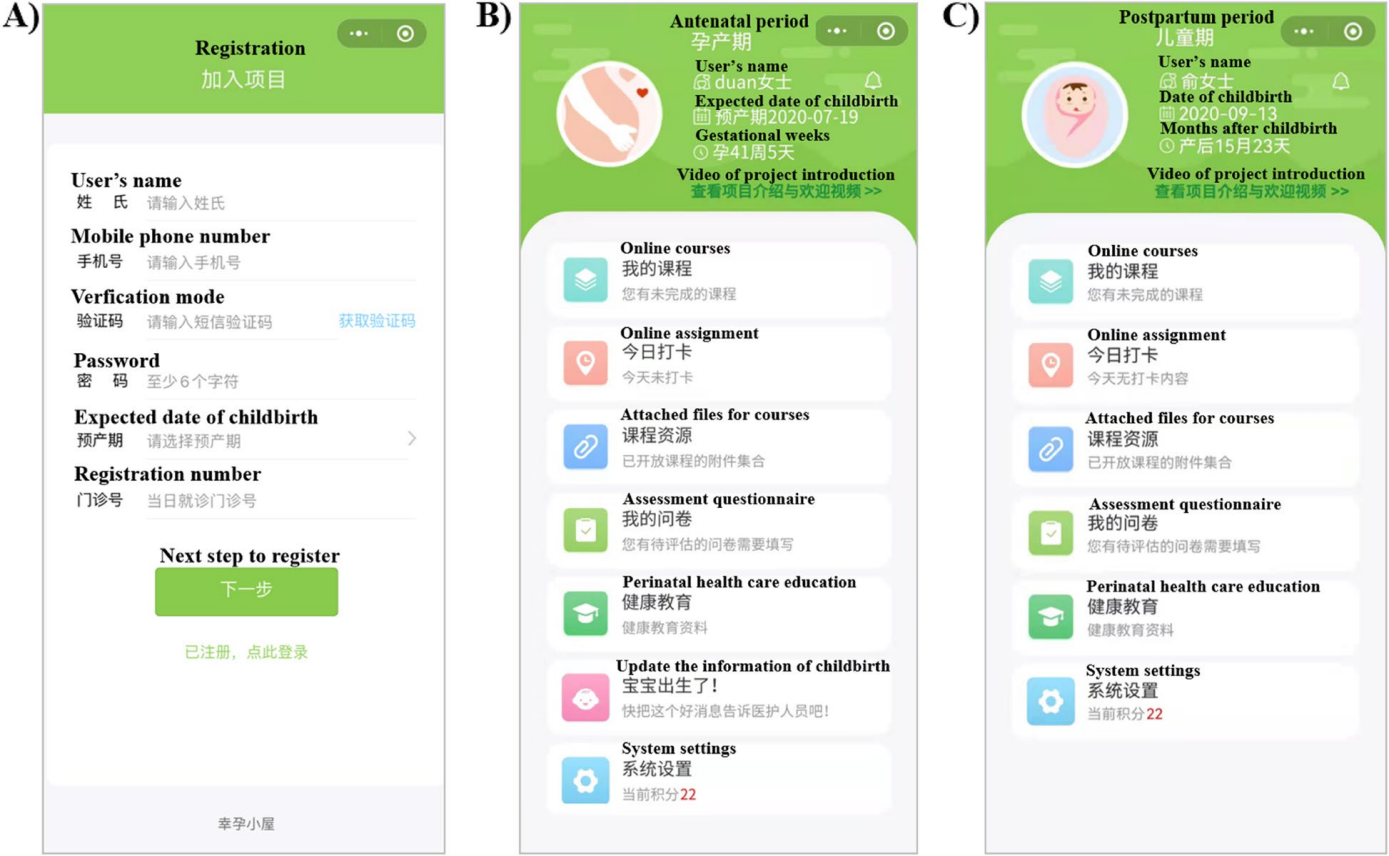
An example of the mobile app providing perinatal mental healthcare. **A** The registration page to sign up. **B** The contents page of providing iCBT treatment during pregnancy. **C** The contents page of providing iCBT treatment during postpartum period

The iCBT intervention will be provided via personal devices connected to the internet or mobile network. A perinatal mental healthcare app has been designed for participants to receive text or video-based psychoeducational modules published weekly for 7 weeks by a trained therapist (Fig. 2). Three modules will be delivered antenatally, and four will be provided postnatally available until 5 weeks after delivery. Weekly modules include psychoeducation, self-monitoring logs and outcome monitoring. Written feedback from participants to the therapist via the app is also required after each module is published. Consistent with well-established CBT theory and protocols [36, 37], the main component of iCBT includes mental health education, cognitive reconstruction, problem solving strategies, behavior reinforcement, and relapse prevention. Table 1 shows the schedule of seven-week period iCBT intervention. The app records metrics that can be used to evaluate adherence or engagement, including completion of modules and recording logs, and duration of time spent in the online system. The study team will have access to the weekly activity logs and be aware of adherence to online modules. Participants will receive an automatic reminder message via the app as well as personal contact from the therapist if they have not engaged with the weekly module material or provided feedback.

**Table 1:** Schedule of ICBT courses

Modules	Time arrangement	Content	Assignment for participants
**During pregnancy**			
Mental health education	The next day after enrolment	Education on pregnancy and mental health, including the impact of pregnancy on maternal mental health and the concept of CBT	Self-evaluation and goal setting
Behavioral activation	The second week after enrolment	Concept of negative behavior, avoidance behavior and procrastination. The use of positive or negative reinforcement	Record positive or negative behavior and make a list of behavior which should be reinforced. Set up a reward system for carrying out a mission
Cognitive restructuring	The third week after enrolment	Relationship between negative automatic thoughts and depression and anxiety. Awareness of cognitive bias and problem solving strategies	Make a list of personal typical negative automatic thoughts and record automatic thoughts within the past week. Find out negative proposals and selective thinking patterns
**Postpartum period**			
Mental health education	2 weeks postpartum	Knowledge of postnatal health care and symptoms of perinatal depression. Review the concept of CBT	Self-evaluation and goal setting
Behavioral activation	3 weeks postpartum	Make use of positive or negative reinforcement	Record positive or negative behavior and make a list of behavior which should be reinforced. Set up a reward system for carrying out a mission
Cognitive restructuring	4 weeks postpartum	Recognition of negative automatic thoughts and strategies to solve the problems	Make a list of personal typical negative automatic thoughts and record automatic thoughts within the past week. Awareness of cognitive bias
Summary and relapse prevention	5 weeks postpartum	Make a summary of what have learned. Provide strategies for preventing relapse	Make a future plan and practice mindfulness
**Additional modules**			
Worry and anxiety	37 weeks of gestation	Problems in regard to pregnancy, labor and caring newborn	Share concerns about labor and childcare and find out ways to solve the problem
Sleeping problem	38 weeks of gestation	Provide problem solving strategies	Record sleeping habits, improve sleeping environment, and restrict time for sleep
Interpersonal relationship	1 week postpartum	The way of communication and psychological education of shifting roles and responsibilities in home	The relationship between parents and understanding the role of a mother. Skills to improve mood and cope with other family members
Mother-infant relationship	1 week postpartum	Psychological support for new mothers	Share feelings and give feedback about difficulties in being a mother

#### Usual care control

Women in both groups will receive standard antenatal depression care including maternal self-care educational videos and mental health assessment. The educational videos include information regarding labor and delivery, postpartum self-care strategies, infant care, and breastfeeding. Women are assessed for depression and anxiety symptoms using the PHQ-9 and GAD-7 respectively at 6 weeks postpartum via the app. Women with a PHQ-9 score > 14 or > 1 in the last PHQ-9 item related to self-harm will be referred to a psychiatrist for further assessment. No concomitant treatments would be prohibited during this trial. However, we will mark participants who have been referred to psychiatrist for drug treatment and complete follow-up data. Analysis would be conducted for all participants enrolled as well as participants excluding those receiving drug treatment.

#### Therapist training and treatment fidelity

Graduate-level trainees of psychology will be iCBT therapists and provided with training and supervision by a registered psychologist with experience in depression management. Training consisting of a 2-day workshop prior to engaging in the intervention and a manual detailing weekly content for iCBT was created to assist therapists. Intervention adherence is defined by proportion of modules completed within 5 weeks postpartum. Intervention engagement will be defined as length of time spent on the modules. This will include (1) frequency of visits to iCBT modules, (2) duration of time spent on each module, and (3) rate of assignment submission.

## Outcomes

Primary and secondary outcomes were chosen based on previous CBT studies and other postpartum depression prevention trials. The aim is to include outcomes that will evaluate effect of the iCBT intervention on depression symptoms that started before childbirth and prevent the development of depression across the first year postpartum. Refer to Table 2 for the schedule of study assessments measured at each follow-up time point to 12 months postpartum.

**Table 2:** Outcome measurement and timing of data collection

Data collection		Time of measurement					
Content	Assessment criteria	Baseline	Postpartum				
			6 weeks	3 months	6 months	9 months	1 year
Demographics	Questionnaire form	**×**					
Primary outcomes							
Depression	PHQ-9		**×**				
Secondary outcomes							
Depression	PHQ-9			**×**	**×**		**×**
Anxiety	GAD-7		**×**	**×**	**×**		**×**
Sleep Quality	PSQI			**×**	**×**		**×**
Social Support	MSPSS				**×**		
Parenting satisfaction	PSI-SF					**×**	
Co-parenting relationship	BCRS					**×**	
Infant development	DDST			**×**	**×**		**×**

## Primary outcome measure

The primary outcome is prevalence of depression at 6 weeks postpartum using the PHQ-9, which is a 9-item self-report measurement used to assess depressive symptoms over the last 2 weeks [27]. Each item is scored from 0 (not at all) to 3 (nearly every day) for a total score ranging from 0 to 27. Scores of ≥ 5, ≥ 10, and ≥ 15 represent mild, moderate, and severe levels of depressive symptoms respectively. Psychometric properties and sensitivity to change are well documented [38]. We will re-administer the PHQ-9 at 12, 24, and 52 weeks postpartum to evaluate the longer-term treatment effect of iCBT and the prevention of new cases of depression.

## Secondary outcome measures

The GAD-7 [28] is a 7-item scale that measures symptoms of anxiety over the last 2 weeks. Items are scored from 0 to 3 where a total score ranges from 0 to 21 and cut-off scores for mild, moderate and severe anxiety symptoms are 5, 10, and 15 respectively.

The PQSI [29] is a 19-item scale that measures sleep quality and disturbances over the past month. Items generate seven “component” scores: subjective sleep quality, sleep latency, sleep duration, habitual sleep efficiency, sleep disturbances, use of sleeping medication, and daytime dysfunction. Total scores range from 0 to 21 where higher scores indicate worse sleep quality. A cutoff score of 5 is also recommended to distinguish good and poor sleep quality with a good sensitivity of 89.6% and specificity of 86.5%.

The MSPSS [30] is a 12-item scale that measures the perceived availability and adequacy of emotional and instrumental social support using a 7-point Likert scale ranging between 1 “very strongly disagree” and 7 “very strongly agree.” Total scores range from 12 to 84 where higher scores indicate higher levels of perceived support. The MSPSS has demonstrated strong internal consistency (Cronbach’s *α* = 0.87–0.94) and test–retest reliability (*r* = 0.73) among adults.

The PSI-SF [31] is a 36-item self-report measure of parenting stress. It includes three subscales: Parental Distress (PD; e.g., “I feel trapped by my responsibilities as a parent”, “I feel lonely and without friends”), Parent-Child Dysfunctional Interaction (PCDI; e.g., “Sometimes I feel my child doesn’t like me and doesn’t want to be close to me”, “When I do things for my child I get the feeling that my efforts are not appreciated” ), and Difficult Child (DC; e.g., “My child makes more demands on me than most children”, “My child gets upset easily over the smallest thing”). Each subscale consists of 12 items rated from 1 (strongly disagree) to 5 (strongly agree), with subscales scores ranging from 12 to 60. A Total score is calculated by summing the three subscales scores, ranging from 36 to 180. Scores of 90 or above may indicate a clinical level of stress.

The BCRS [32] is a 14-item self-report measure of co-parenting and consists of 7 subscales: co-parenting agreement, co-parenting closeness, exposure to conflict, co-parenting support, co-parenting undermining, endorse partner parenting, and division of labor. Each subscale involves questions including 7 options ranging from 0 (never) to 6 (very often) or 0 (not true of us) to 6 (very true of us). Lower scores in the subscales of exposure to conflict and co-parenting undermining indicate more positive co-parenting as does higher scores in the remaining five subscales.

The DDST [33] is a scale of developmental assessment covering the ages from birth to 6 years. It is made up of 105 items grouped into four sectors including gross motor, fine motor-adaptive, language, and personal-social. Each item is represented by a bar that spans the ages at which 25%, 50%, 75%, and 90% of typically developing children in the standardization sample passed that item. Items that over 90% of children should be able to accomplish which are not successfully completed are considered as a delay and items where the age line passes through the 75–90% section of the bar that are not completed are scored as caution. The results are categorized into 3 types: normal, abnormal (two or more delays), and questionable (two cautions or one delay).

## Sample size and statistical analysis

Based on a previous trial, Chinese pregnant women with depressive symptoms who received CBT delivered face-to-face showed significantly lower rates of postpartum depression than those who received standard antenatal care (22.2% vs 47.3%, *P* = 0.001) [25]. We estimate that a sample size of at least 178 (89 per group, assuming 20% loss to follow-up) is required to detect a 25% reduction in depression symptoms at 6 weeks postpartum on based on a 90% power to detect differences between groups and a two-sided significance level of 0.05. Considering the possible decreased efficacy due to CBT delivered by internet rather than face-to-face, we aim to recruit 300 participants in this study.

All data will be extracted from the security server of the main center and incorporated in a single Microsoft Excel database through the computer system. The original data will be verified by researchers before analyzed and non-conforming data will be excluded with certain reasons listed. For analysis, descriptive statistics of continuous variables will be represented as means and standard deviations or medians and inter quartile ranges. Categorical variables will be expressed as frequencies with proportions. Differences of continuous variables with normal distribution between groups will be tested by Student’s *t*-test, whereas Mann-Whitney *U* test will be applied to variables with skewed distribution. Categorical variables will be tested by chi-square test. Demographic and medical characteristics at baseline will be analyzed for whether they are equivalent between two groups; otherwise, a subgroup analysis will be performed for adjusting confounding factors. Main analyses will be conducted using intention-to-treat (ITT) model, in which all participants will be included based on random allocation regardless of study completion. For outcomes collected at multiple time points, repeated measures design using generalized estimating equation (GEE) models will be conducted to fit outcomes that are measured at each time point, so that all the participants might contribute to the analyses, even if there were missing data at some of the follow-up points. In addition, statistical analysis of the baseline characteristics of those who remain in the study and those who are lost to follow-up will also be conducted to explore whether there is differential drop out. All statistical analyses will be performed using the R software (version 4.0.2) or other statistical software packages if necessary. Differences will be considered significant if the two-sided p value is less than 0.05.

## Risk management strategies

Several strategies will be implemented to mitigate potential risk. First, potential participants who endorse self-harm on the EPDS or PHQ-9 undergo a suicide risk assessment. Those who score positive for suicide ideation will be referred to the emergency department or a mental health specialist for immediate assessment. As discussed in the consent form to ensure safety, the emergency department or a mental health specialist will be contacted on the participant’s behalf if necessary. Considering the intervention is a non-pharmacological treatment, adverse effects due to the intervention are negligible. However, severe adverse events including suicidal behaviors or deaths will be recorded in case report form and reported in the future published manuscript. Participants are allowed to leave the study at any time based on their personal willingness. Research assistants are required to complete the withdrawal report form in detail.

## Ethics and dissemination

Ethical approval was obtained from the institutional review board of International Peace Maternity and Child Health Hospital (GKLW2020-25). All substantial amendments will be notified to the research ethics committee and to the regulatory authority. Results from this trial will be disseminated to the academic community through conference presentations and the publication of peer-reviewed manuscripts. Results will further be made available to participants, health care providers, and the general public. For further access to participant-level dataset or statistical code, please contact the corresponding author.

## Data management

Baseline data including demographic characteristics and pregnancy complications will be collected by the research assistant using an online questionnaire based on previous research conducted by our team [6]. The name of each participant will be replaced with a computer-generated serial number for identification. All data will be stored on a secure server at the International Peace Maternity and Child Health Hospital and will be password-protected. Only the principal investigator will have access to any identifiable data, and the data will be analyzed by a biostatistician blinded to group allocation. De-identified data will be made available on reasonable request if compliant with the receipt of ethical approval from both the sending and receiving institutional ethics review boards.

## Patient and public involvement

Women with lived experience were consulted in the design of this project and assisted in preparation of study materials. Engagement will continue throughout trial conduction and be emphasized when preparing materials for dissemination.

## Research team structure

Structure of the research team consists of four components including the expert committee, the executive team, the medical information department, and the data monitoring committee. The expert committee is composed of obstetricians, psychiatrists, and statisticians who are responsible for designing the clinical research trial and online course modules. They are also in charge of drafting a manual of standard operating procedure and training members of the executive team. The executive team comprises research assistants from the participating hospitals who are responsible for screening and recruiting participants, obtaining signed informed consent, collecting baseline data, and randomizing participants. They are also required to follow participants and provide referral to the psychiatrist if necessary. Members of the medical information department are responsible for system management, app updates, and setting online courses and assignment as well as solving urgent technical problems. An independent data monitoring committee is established for quality and safety control of the study. Members are required to quarterly review data during the trial, oversee research assistant activities, and assess safety of the treatment for adverse events.

## Implications

Faced with increased risks of psychological distress during the COVID-19 pandemic and higher rates of depression and anxiety among pregnant women specifically, there is an urgent need for the development of effective and easily accessible treatment options. Here, we describe the protocol for a RCT that aims to evaluate the efficacy of iCBT in the treatment of antenatal depression with the goal of preventing depressive symptoms across the first year postpartum. Compared with the traditional face-to-face psychological treatment, iCBT addresses many treatment and help-seeking barriers and may be an effective treatment for antenatal depression during a major public health emergency. To date, most studies examining iCBT among depressed pregnant or postpartum women have demonstrated efficacy in a small scale with a limited follow-up period and no assessment of infant outcomes [24, 25, 39, 40]. To overcome these limitations, we plan to recruit 300 pregnant women with depressive symptoms from five diverse settings and will follow them to 1 year postpartum to evaluate longer-term impact on both women and their infants. If positive results from this trial are obtained, it will provide strong evidence for the effectiveness of iCBT in the treatment of antenatal depression with the goal of preventing the development of depression across the first year postpartum. Furthermore, if proven effective, this intervention has the potential that can be provided during major public health emergencies.

## Supplementary Information

**Additional file 1.**

## Abbreviations

CBT: Cognitive behavioral therapy
EPDS: Edinburgh Postnatal Depression Scale
COVID-19: Coronavirus disease 2019
PHQ-9: Patient Health Questionnaire 9-item scale
GAD-7: Generalized anxiety disorder
PSQI: Pittsburgh Sleep Quality Index
MSPSS: Multidimensional Scale of Perceived Social Support
PSI-SF: Parenting Stress using Parenting Stress Index-Short Form
BCRS: Brief Co-parenting Relationship Scale
DDST: Denver Development Screen Test
