# Supplementary material for: Internet-based cognitive therapy for women with antenatal depressive symptoms during the COVID-19 pandemic: protocol for a multi-center randomized controlled trial across China
Source: Trials. 2022 Sep 21;23:797. doi: 10.1186/s13063-022-06728-5 (PMC9490680; doi:10.1186/s13063-022-06728-5)
Supplement: Supplementary file 1 — Additional file 1. [file 13063_2022_6728_MOESM1_ESM.pdf]

Supplementary material information

Title: Internet-based cognitive therapy for women with antenatal depressive symptoms during the COVID-19 pandemic: protocol for a multi-center randomized controlled trial across China

Included files:

SPIRIT Checklist

Model consent form

SPIRIT 2013 Checklist: Recommended items to address in a clinical trial protocol and related documents*

Section/item	Item No	Description	Addressed on page
**Administrative information**			
Title	1	Descriptive title identifying the study design, population, interventions, and, if applicable, trial acronym	P3
Trial registration	2a	Trial identifier and registry name. If not yet registered, name of intended registry	P4
	2b	All items from the World Health Organization Trial Registration Data Set	NA
Protocol version	3	Date and version identifier	2020715-version1.0
Funding	4	Sources and types of financial, material, and other support	P16
Roles and responsibilities	5a	Names, affiliations, and roles of protocol contributors	P7,P15-16
	5b	Name and contact information for the trial sponsor	Model consent form in Supplementary material (P11)
	5c	Role of study sponsor and funders, if any, in study design; collection, management, analysis, and interpretation of data; writing of the report; and the decision to submit the report for publication, including whether they will have ultimate authority over any of these activities	P16

	5d	Composition, roles, and responsibilities of the coordinating centre, steering committee, endpoint adjudication committee, data management team, and other individuals or groups overseeing the trial, if applicable (see Item 21a for data monitoring committee)	P15
**Introduction**			
Background and rationale	6a	Description of research question and justification for undertaking the trial, including summary of relevant studies (published and unpublished) examining benefits and harms for each intervention	P6, P15
	6b	Explanation for choice of comparators	P8, P10
Objectives	7	Specific objectives or hypotheses	P7
Trial design	8	Description of trial design including type of trial (eg, parallel group, crossover, factorial, single group), allocation ratio, and framework (eg, superiority, equivalence, noninferiority, exploratory)	P7, Figure 1
**Methods: Participants, interventions, and outcomes**			
Study setting	9	Description of study settings (eg, community clinic, academic hospital) and list of countries where data will be collected. Reference to where list of study sites can be obtained	P7
Eligibility criteria	10	Inclusion and exclusion criteria for participants. If applicable, eligibility criteria for study centres and individuals who will perform the interventions (eg, surgeons, psychotherapists)	P7-8
Interventions	11a	Interventions for each group with sufficient detail to allow replication, including how and when they will be administered	P9-12

	11b	Criteria for discontinuing or modifying allocated interventions for a given trial participant (eg, drug dose change in response to harms, participant request, or improving/worsening disease)	P13
	11c	Strategies to improve adherence to intervention protocols, and any procedures for monitoring adherence (eg, drug tablet return, laboratory tests)	P10
	11d	Relevant concomitant care and interventions that are permitted or prohibited during the trial	P9-10
Outcomes	12	Primary, secondary, and other outcomes, including the specific measurement variable (eg, systolic blood pressure), analysis metric (eg, change from baseline, final value, time to event), method of aggregation (eg, median, proportion), and time point for each outcome. Explanation of the clinical relevance of chosen efficacy and harm outcomes is strongly recommended	P10-12
Participant timeline	13	Time schedule of enrolment, interventions (including any run-ins and washouts), assessments, and visits for participants. A schematic diagram is highly recommended (see Figure)	Table 2
Sample size	14	Estimated number of participants needed to achieve study objectives and how it was determined, including clinical and statistical assumptions supporting any sample size calculations	P12-13
Recruitment	15	Strategies for achieving adequate participant enrolment to reach target sample size	P8

Methods: Assignment of interventions (for controlled trials)

Allocation:

Sequence generation	16a	Method of generating the allocation sequence (eg, computer-generated random numbers), and list of any factors for stratification. To reduce predictability of a random sequence, details of any planned restriction (eg, blocking) should be provided in a separate document that is unavailable to those who enrol participants or assign interventions	P8
Allocation concealment mechanism	16b	Mechanism of implementing the allocation sequence (eg, central telephone; sequentially numbered, opaque, sealed envelopes), describing any steps to conceal the sequence until interventions are assigned	P8
Implementation	16c	Who will generate the allocation sequence, who will enrol participants, and who will assign participants to interventions	P8,P15
Blinding (masking)	17a	Who will be blinded after assignment to interventions (eg, trial participants, care providers, outcome assessors, data analysts), and how	P8
	17b	If blinded, circumstances under which unblinding is permissible, and procedure for revealing a participant's allocated intervention during the trial	P8
**Methods: Data collection, management, and analysis**			
Data collection methods	18a	Plans for assessment and collection of outcome, baseline, and other trial data, including any related processes to promote data quality (eg, duplicate measurements, training of assessors) and a description of study instruments (eg, questionnaires, laboratory tests) along with their reliability and validity, if known. Reference to where data collection forms can be found, if not in the protocol	P11-12

	18b	Plans to promote participant retention and complete follow-up, including list of any outcome data to be collected for participants who discontinue or deviate from intervention protocols	P9
Data management	19	Plans for data entry, coding, security, and storage, including any related processes to promote data quality (eg, double data entry; range checks for data values). Reference to where details of data management procedures can be found, if not in the protocol	P14
Statistical methods	20a	Statistical methods for analysing primary and secondary outcomes. Reference to where other details of the statistical analysis plan can be found, if not in the protocol	P12-13
	20b	Methods for any additional analyses (eg, subgroup and adjusted analyses)	P13
	20c	Definition of analysis population relating to protocol non-adherence (eg, as randomised analysis), and any statistical methods to handle missing data (eg, multiple imputation)	P13
**Methods: Monitoring**			
Data monitoring	21a	Composition of data monitoring committee (DMC); summary of its role and reporting structure; statement of whether it is independent from the sponsor and competing interests; and reference to where further details about its charter can be found, if not in the protocol. Alternatively, an explanation of why a DMC is not needed	P15
	21b	Description of any interim analyses and stopping guidelines, including who will have access to these interim results and make the final decision to terminate the trial	NA. There will be no interim analysis in this study.

Harms	22	Plans for collecting, assessing, reporting, and managing solicited and spontaneously reported adverse events and other unintended effects of trial interventions or trial conduct	P13
Auditing	23	Frequency and procedures for auditing trial conduct, if any, and whether the process will be independent from investigators and the sponsor	P15
**Ethics and dissemination**			
Research ethics approval	24	Plans for seeking research ethics committee/institutional review board (REC/IRB) approval	P3
Protocol amendments	25	Plans for communicating important protocol modifications (eg, changes to eligibility criteria, outcomes, analyses) to relevant parties (eg, investigators, REC/IRBs, trial participants, trial registries, journals, regulators)	P14
Consent or assent	26a	Who will obtain informed consent or assent from potential trial participants or authorised surrogates, and how (see Item 32)	P8
	26b	Additional consent provisions for collection and use of participant data and biological specimens in ancillary studies, if applicable	NA. There will be no collection and use of participant data and biological specimens for ancillary studies.
Confidentiality	27	How personal information about potential and enrolled participants will be collected, shared, and maintained in order to protect confidentiality before, during, and after the trial	P14
Declaration of interests	28	Financial and other competing interests for principal investigators for the overall trial and each study site	P16
Access to data	29	Statement of who will have access to the final trial dataset, and disclosure of contractual agreements that limit such access for investigators	P17

Ancillary and post-trial care	30	Provisions, if any, for ancillary and post-trial care, and for compensation to those who suffer harm from trial participation	NA. The intervention of our study is an online education module, which might hardly induce any harm for participants, and we also offer financial incentives for each participant. See P8 and model consent form for information on financial incentives.
Dissemination policy	31a	Plans for investigators and sponsor to communicate trial results to participants, healthcare professionals, the public, and other relevant groups (eg, via publication, reporting in results databases, or other data sharing arrangements), including any publication restrictions	P4
	31b	Authorship eligibility guidelines and any intended use of professional writers	P16
	31c	Plans, if any, for granting public access to the full protocol, participant-level dataset, and statistical code	Available by request
**Appendices**			
Informed consent materials	32	Model consent form and other related documentation given to participants and authorised surrogates	Model consent form in Supplementary material
Biological specimens	33	Plans for collection, laboratory evaluation, and storage of biological specimens for genetic or molecular analysis in the current trial and for future use in ancillary studies, if applicable	NA. No biological specimen will be collected in this study.

*It is strongly recommended that this checklist be read in conjunction with the SPIRIT 2013 Explanation & Elaboration for important clarification on the items. Amendments to the protocol should be tracked and dated. The SPIRIT checklist is copyrighted by the SPIRIT Group under the Creative Commons "Attribution-NonCommercial-NoDerivs 3.0 Unported" license.

Informed Consent

Dear expectant mothers:

We are sincerely inviting you to participate in a project to promote the antenatal and postpartum mental health in women. The project aims to evaluate the physiological and mental health of women during perinatal and puerperium period, so as to further improve the maternal and child health care system and safety management, as well as to reduce the incidence of adverse pregnancy and neonatal outcomes by establishing a maternal mental health care system invloving prenatal screening for high risks of depression, providing internet-based cognitive behavior therapy and postnatal psychological evaluation. It is necessary to understand the purpose and content of the study before you decide to participate. Please read the following items carefully and discuss it with your doctor, family and friends. If you have any questions or want more relevant information, please consult with your doctor or contact the staff listed at the end of the informed consent.

What is the purpose of the study?

Women undergo a series of physiological and psychological changes during pregnancy, childbirth, postpartum recovery and lactation, leading to high risks of mental disorders. Perinatal mental disorders might not only affect the physical and psychological health of pregnant women, but might also lead to pregnancy complications, adverse neonatal outcomes, and cognitive behavioral abnormalities in offspring. However, screening and treatment for antenatal depression has not been taken as a routine clinical practice in China, and has not been taken seriously by specialists including obstetricians. Therefore, this project is carried out to establish a maternal mental health care system invloving prenatal screening for high risks of depression, internet-based cognitive behavior therapy and postnatal psychological evaluation in several hospitals nationwide for the first time, so as to provide evidence-based practice for clinical guidelines, and guarantee health and safe of the mother and infant.

Why are you suitable to participate in this study?

You are invited to participate in the study because you meet the following criteria:

You are singleton pregnancy, 18 years of age or older, planning to deliver at this partcipated hospital, able to read and understand Chinese, have reliable internet access via a smartphone or computer, and potentially at a risk of perinatal depression after prenatal screening in the third trimester of pregnancy.

Are there risks associated with participating in the study?

If you agree to participate in the study, you will be invited to cooperate in online follow-up at the third trimester of pregnancy, and 6 weeks, 3 months, 6 months, and 1 year postpartum to evaluate your mental health and newborn development.

You will also be invited to participate in several courses, which will be published item by item online from the third trimester to 4 weeks postpartum.

The research team will follow up until 1 year postpartum, and contact you for further assessment if necessary to prevent more serious health problems. There will be no adverse effect on your daily work, routine medical practice, and the health of you and your baby.

Will there be any cost or compensation?

There is no additional cost for you to participate in the study. In additon, you can earn points by completing your online courses and questionnaires, which can be exchanged for cash at the end of the study (note: the maximum amount is 500 RMB).

Will my information be confidential?

During the research, we will keep all your personal information confidential. Only monitors of the research team have access to your medical records in order to check whether the information is collected reliably and ensure that the study is carried out properly.

All data will be transferred to the security server of International Peace Maternity and Child Health Hospital after coded. The code used to identify you will not be revealed to anyone except for the authorized personnel in the research team. All information in the computer will be protected by setting a password.

The results of the study might be reported at medical conferences and published in scientific journals. Only non-identifiable information will be used for presentation and public publication. All data will be presented as population data, not individual data.

Do I have to participate?

Participation in the study is not forcible, but entirely voluntary. You can also withdraw at any time during the research without any reason. Whatever your decision is, it will not affect your normal treatment or your relationship with your health care providers.

Who should I contact for more information?

If you have additional questions or concerns after reading this informed consent and discussing with your doctor, please contact us as following way:

Trial sponsor: Professor He-Feng Huang

Phone number: 86-21-64070434

Address: No. 910, Hengshan Road, Xuhui District, Shanghai

Who approved the study?

This study was approved by the Medical Research Ethics Committee of the International Peace Maternity and Child Health Hospital (GKLW2020-25).

I hereby agree to participate in the study.

Participant's name: ___________________________

Participant's signature: ___________________________

Family member's signature: ___________________________

Researcher's signature: ___________________________

Date: ___________________________

知情同意书

亲爱的准妈妈们，您好！

我们真诚地邀请您参加一项促进孕产妇围产期及产褥期心理健康的项目，旨在通过建立围产期抑郁高危筛查—基于互联网的线上认知行为干预—产后评估的心理健康维护体系，更全面地了解并评估孕产妇围产期及产褥期的生理心理健康状况，进一步完善母婴保健体系与安全管理工作，降低围产期及产后不良并发症的发生率。在您决定参加之前，了解该研究的目的和内容是非常必要的，请您认真阅读以下内容，并可与您的医生、家人、朋友讨论。如您有任何疑问，或想了解更多相关信息，请向医生咨询或直接与文末所列工作人员联系。

该研究的目的是什么？

女性在经历妊娠、分娩、产后恢复及哺乳时将发生一系列的生理和心理变化，这一阶段使女性处于情绪障碍的高风险中，不仅仅影响孕产妇的身心健康，产前和产时的不良情绪也可能对分娩方式、产程、产后并发症以及新生儿预后、子代情绪、行为、智力和认知能力等造成不同程度的不良影响。然而目前我国的医疗机构尚未将围产期情绪管理工作作为常规孕产妇保健项目，包括妇产科医师在内的医务人员对于孕产妇的情绪调理缺乏重视。因此本项目拟首次在全国范围内的数家医院建立孕产妇围产期抑郁高危筛查—线上认知行为干预—产后评估的心理健康维护体系，从而指导临床治疗方案的优化，促进未来临床应用指南的制定，保障孕产妇和婴儿的身心健康。

您为什么适合参与该研究？

我们邀请您参加该研究是因为您符合以下情况：

您计划在参加研究的医院分娩，并正处于妊娠晚期，能够阅读、理解知情同意书，经过产前筛查存在发生围产期抑郁的潜在风险，符合本项研究的目标人群标准。

参与研究有危险吗？

如果您同意参加该研究，我们将请您配合参与随访，分别在孕晚期、产后 6 周、3 个月、半年及一年阶段，随访内容主要对您的情绪、生活方式以及新生儿的发育情况进行评估，随访方式通过基于互联网的线上实行。

如果您参与了健康教育组，您将被邀请参与相关课程学习，课程将通过互联网自孕晚期至产后 4 周逐项发布。

研究小组将对您随访情况密切关注并实时评估，必要时将与您取得联系，以尽可能防止更严重的健康问题，但对您的日常生活工作、正常诊疗流程、您与宝宝的身体健康没有任何影响。

需要花销或有报酬吗？

参与该项研究，您不需要花费额外费用。根据您课程学习和随访问卷的完成情况，您可以获得相应积分，在研究结束时可根据积分兑换相应数额的现金（注：最高金额为 500 元）。

我的信息是保密的吗？

研究过程中，我们会严格保密关于您的所有信息。只有相关医院工作人员才有权查看您的医疗档案，以便核查所收集信息的准确性，确保研究工作正常进行。

任何以电子形式传输的信息都将重新编码加密后传输至国际和平妇幼保健院的安全服务器。

用于识别您的编码将不会透露给研究团队内授权人员以外的人，计算机中的所有信息都将通过设置密码加以保护。

该研究的结果可能会汇报于医学会议和发表于科技杂志上，但不会使用任何可识别至您个人的相关信息。所有数据将以人群数据的形式呈现，而非个体数据。

我必须参加吗？

参与该研究应完全出于自愿，而不是被迫参加。参加研究的过程中也可随时退出，无需任何理由。不管您的决定是什么，都不会影响您的正常诊治或您与医护人员的关系。

如需要更多的信息，我应该和谁联系？

在阅读完该介绍并与您的医生讨论后，如果您还有其他疑问或顾虑，请与以下工作人员联系:

研究人员：黄荷凤 教授

电话号码：86-21-64070434

地址：上海市徐汇区衡山路 910 号

谁批准了该研究的进行？

本研究已由以下伦理委员会批准：中国福利会国际和平妇幼保健院伦理委员会

我在此同意参加该研究。

参与者姓名（印刷体填写）：吴晓燕

参与者签名：吴晓燕

参与者家属签名：________________

研究人员签名：[illegible]

日期：2020 年 12 月 10 日
